# Housing investment and family entrepreneurship: Evidence from China

**DOI:** 10.1371/journal.pone.0285699

**Published:** 2023-06-02

**Authors:** Xiaobing Huang, Liu Min, Xinxin Meng, Xiaolian Liu, Yousaf Ali Khan, Syed Zaheer Abbas

**Affiliations:** 1 School of Business, Gannan Normal University, Ganzhou, China; 2 School of Economics and Management, Gannan Normal University, Ganzhou, China; 3 Department of Mathematics and Statistics, Hazara University Mansehra, Mansehra, Pakistan; Universiti Kebangsaan Malaysia, MALAYSIA

## Abstract

The aim of this article is to explore the impact of housing investment on household entrepreneurship. Using survey data from China and employing a Probit model, we examine three aspects of housing status and innovatively subdivide household entrepreneurship into agricultural entrepreneurship and business entrepreneurship. The results show that households with higher housing investment are less likely to become agricultural entrepreneurs, but more likely to start a new business. Households with full-owned housing enjoy a higher likelihood to become business entrepreneurs. However, other ownerships have no relation with the choice of entrepreneurship. More housing loans discourage entrepreneurial activities. One exception is that bank loan raises the chance of being agricultural entrepreneurs. Households who build their own houses have a higher agricultural entrepreneurship. Buying market price houses encourages households to be business entrepreneurs. Low-price house and inherited house prevent households from being business entrepreneurs.

## 1 Introduction

Since the reform and opening-up, China’s economic development has entered a "new normal" stage after nearly 40 years of rapid growth. The fundamental characteristic of this stage is that economic growth shifts from high-speed model to high-quality model in order to achieve sustainable growth. However, in the short run, the slowdown of the economic development inevitably results in some adverse consequences. For instance, the deceleration of economic development will cause stronger employment pressure, especially with the increasing number of college graduates and rural-urban migrants. How to create more jobs is a great challenge for the government in recent years? There is no doubt that entrepreneurship is the engine of economic growth and a solution for employment in developing countries [1, 2].

Since the concept of "Mass entrepreneurship and innovation" was proposed by Premier Li at the 2014 Summer Davos in Tianjin, lots of efforts have been put by Chinese government to promote entrepreneurship to a higher level. These efforts have delivered positive results. The number of entrepreneurial teams and innovation-driven companies have achieved fast growth. A daily average of 18,000 new businesses have been registered in 2018. Nevertheless, there is no denying that compared with China economic development and huge employment demand, entrepreneurship has obvious "shortcomings" in terms of quantity, quality and environment. Regarding quantity, according to World Bank statistics, the density of newly registered enterprises in China (the number of newly registered enterprises per 1,000 working-age population) is less than 2, which is lower than 28.12 in Hong Kong, 8.04 in Singapore and 12.16 in Australia. College students should be the main force of entrepreneurial activities, but in China, the proportion of college students in entrepreneurship only accounts for about 1% of the total number of graduates, while in developed countries, this proportion is about 20% -30%. In terms of quality, related surveys show that the overall success rate of entrepreneurship in China is relatively low. Less than 10% of entrepreneurs can successfully start their own businesses, and less than 10% of newly founded enterprises can survive more than one year. In addition, the entrepreneurial success rate of Chinese college students is even lower. According to “2017 China College Student Employment Report”, more than half of the graduates who started their own businesses half a year after graduation quit their businesses in three years. Even in coastal cities with a better entrepreneurial environment, such as Zhejiang and Guangdong, the entrepreneurial success rate of college students is only about 5%, while the global average success rate is almost 20%.

Moreover, with respects to the type of entrepreneurial activity, the proportion of technological entrepreneurship in China is relatively small, which also suggests a low quality of entrepreneurship. According to the Global Entrepreneurship Watch (GEM) 2018/2019 China Report, the proportion of Chinese technology entrepreneurs is 2.66%, which is far behind the top economies such as Australia (13.1%), the United Kingdom (11.27%) and Japan (10.58%). The entrepreneurial activities mainly concentrated in the service industry in China, including wholesale and retail, which accounts for more than 60% of the total entrepreneurial activities. In terms of the entrepreneurial environment, the Global Entrepreneurship Watch (GEM) 2018/2019 China Report also show that, although the overall score of China’s entrepreneurial environment is 5.0, ranking 6th among G20, China obtained the lowest scores in business and legal infrastructure. Still there is much room for progress in China’s entrepreneurial environment, especially in entrepreneurship education, R&D transfer, and business and legal infrastructure.

The high importance of entrepreneurship placed by governments brings closer academic attentions worldwide to this topic in several directions. More attention is paid to the study of the factors that affecting entrepreneurial activities. Some of them took demographic factors into consideration, such as gender [3], age [4, 5], Education [6, 7]. Others focused the financial factors, for instance, household wealth [8–10], housing price [11, 12]. The rest of them kept an eye on institutional factors, such as government regulation [13], corruption [14] and social security [15].

Entrepreneurial activities require financial support, but most of the household capital in China is concentrated in housing assets, so housing assets largely influence household entrepreneurial behavior. However, housing has both asset and investment attributes, so there is also a positive "wealth effect", " collateral effect" and negative "crowding-out effect" on household entrepreneurial activity at the same time " [16–19]. The positive effect is mainly reflected in the fact that housing has a certain value that can be transformed into cash, whether it is sold or mortgaged, to provide financial support for household entrepreneurial activities. The negative effect is mainly reflected in the fact that the purchase of housing takes away from the entrepreneurial capital, especially due to the influence of marriage customs, Chinese urban households generally prefer to purchase housing and reduce entrepreneurial activities. At the same time, in times of rising housing prices, households may choose higher-return housing investments over entrepreneurial activities that carry greater risks. Thus, the impact of housing assets on households’ entrepreneurial choices is uncertain.

However, little is known about the nexus between housing and entrepreneurship. As a single biggest asset in most households’ balance sheets, housing has a significant impact on financial decision-making [20]. In order to fill the gap of previous research, this study investigates the impact of housing investment on the household entrepreneurship by using the data from China Household Finance Survey (CHFS) in 2015.

The contribution of this paper is twofold. First, compared with previous studies, we note that households in Western countries mainly finance their home purchases through bank loans, while Chinese households borrow funds for home purchases not only from bank loans, but also from private lending. Therefore, private lending should not be ignored in the discussion of Chinese family housing. And the cost of housing varies by access method, and the impact on family entrepreneurship may vary. So we assess the housing situation from different perspectives, including housing holding, housing ownership, housing loan and housing acquisition, which enable us to have a panoramic view over the relation between housing and entrepreneurship. Moreover, unlike previous studies, the paper distinguishes entrepreneurial activities into business entrepreneurship and agricultural entrepreneurship, which gives our research more policy implications.

The rest of this paper is organized as follows. Sector 2 reviews relevant literature. Section 2 introduces the history of housing reform in China. Section 4 introduces the data and specifications, defines and statistically describes the variables. Section 5 shows and explains the empirical results including benchmark results and robustness checks. The last section concludes the study and delivers policy implications.

## 2 Literature review

Housing is not only used for inhabitation, but also a popular product to be invested. with the soaring price of housing worldwide, housing has gradually become the biggest assets in most households’ balance sheets. In China, real estate accounts for nearly 70% of total household assets according to 2018 China Family Wealth Survey Report [21]. In US, residential property represents 60% of all personal wealth in the US [22]. Plenty of papers have suggested that potential entrepreneurs can be discouraged from starting a business when facing financial constraints [23, 24]. As a result, the connection between housing and entrepreneurship has also attracted many scholars’ attention [25–28]. The existing literature has explored several mechanisms how housing may affect entrepreneurial participation.

The first channel is wealth effect. Owning a house is a way to store wealth, and this kind of wealth will appreciate as the house price rises. Potential entrepreneurs who owning houses could extract the additional home equity in their property and invest it in their business. Most studies do find a positive relationship between housing wealth and entrepreneurship [29–31]. However, rising housing price could also have a negative impact on entrepreneurial activities, because it may be more attractive to buy a house than start a business when the house price is rising [32].

The second channel is collateral effect. The potential role of the collateral channel of housing in entrepreneurship is clearly understood. Debt financing is the main form of external finance for new business establishers [33, 34], but the amount a small business can borrow isn’t directly observable due to the asymmetric information. Pledging personal collateral, in particular their housing wealth, can facilitate the lending process, and thus increase the likelihood of receiving bank loans [35].

The final channel is called crowding-out effect. A growing number of literature documents that owning a house may negatively affect labor market outcomes. Oswald (1996) found that the increased rate of home ownership could be associated with a lower labor mobility and fewer business opportunities [36]. Most people purchase houses by mortgage rather than lump-sum payment. homeowners paying installment are more likely to be locked in their jobs to avoid the threat of execution, and less likely to take any risks to start a new business [25, 37].

## 3 Background: China’s housing reform

The urban housing system has undergone dramatic changes since the founding of new China in 1949 [38]. Before 1978, as an important part of central planning system, the production, allocation, and pricing of urban housing were controlled by government through the work units. Housing was freely distributed as a welfare according to the seniority, merits, and needs of the employees. Housing is public-owned and not allowed to be sold [39].

The old housing system was reformed since the economic reform took place. In 1988, the commercialization and privatization of urban public housing were executed by government to stimulate home ownership. A great many public housing was sold to employees in work units at very low prices. In 1998, the welfare-based housing system was terminated and the market-based housing system was established [40]. Households were allowed to purchase housing in the real estate market. Banks begun to offer mortgage to the housing buyers. Meanwhile, public rental housing constructed by government was still available to low-income families with low rent. with the economic development and rapid urbanization, a booming housing market developed in China.

After two successful housing reforms, housing has gone from having only residential attributes to having both asset and investment attributes. On the one hand, the real estate market has boomed rapidly and its return on investment is often higher than that of other investment options. This attracted a large influx of capital into the housing market, which weakened the public’s willingness to start businesses and entrepreneurial activities. On the other hand, housing prices continue to rise, and housing with both asset and investment attributes greatly enhances households’ ability to access monetary capital. Thus, both the positive promotion and negative crowding out of entrepreneurial choices by housing investment may be stronger, while the total effect is uncertain and its net effect becomes an empirical question.

## 4 Data, specification and description

### 4.1 Data

This paper uses the data of 2015 China Household Finance Survey (CHFS) collected by the Survey and Research Center for China Household Finance from 29 provinces in China with nearly 40,000 household samples. The use of this questionnaire data is based on two main points. First, the questionnaire provides a very detailed and rich survey on Chinese households’ entrepreneurship, housing investment, which is in line with the research theme of this paper. Second, the survey is comprehensive, representative, with a large sample size and not random. It is now recognized by the academic community as a kind of authoritative data with scientific research value. We filtered the data according to the research needs of this paper using Stata software. After eliminating missing values and extreme values of key variables, we obtain a sample with 32986 households which accounts for about 81% of the original data.

### 4.2 Specification

In order to investigate the connection between housing and entrepreneurship, we employ a Probit model to estimate the effect with the following specification.

$$\text{Pr}\left(Entrepreneurship=1\right)=G(\beta_{0}+\beta_{1}housing+\gamma X)$$

Where the dependent variable *Entrepreneurship* represents the entrepreneurial intention. we distinguish entrepreneurial activities into business entrepreneurship and agricultural entrepreneurship. Reference to previous literature [41], in the questionnaire, the item " Is your family engaged in production and operation of industry and commerce, including individual business, leasing, transportation, online stores, and enterprises" is employed to identify the business entrepreneurship. So that the dependent variable is a dummy equal 1 if the respondent answers YES, otherwise 0.

Guo and Ding (2013) pointed out that agricultural entrepreneurship refers to the process of economic activities related to agriculture by farmers relying on family organizations by investing in productive capital [42]. According to this definition, the item “To which of the following operating types does your family business belong” is adopted to identify the agricultural entrepreneurship. Five options are listed including (1) agricultural enterprises, (2) agricultural cooperatives, (3) family farms, (4) leading specialized households, (5) general rural household. We define the former four options as the agricultural entrepreneurship. So that the dependent variable of core interest is a dummy equal 1 for respondent belongs to the former four types, otherwise 0.

Where the core independent variable *housing* denotes the housing situation of households. We use housing holding, housing ownership, home loan and housing acquisition to evaluate the housing condition. First, the homeownership is described with 3 variables including a dummy equal to 1 if a household owns a house or more, otherwise 0, the number of owned-houses, and the ratio of housing assets measured with market value to total assets. Second, regarding the form of property rights, the questionnaire reports 4 types of housing ownership including full ownership, partial ownership, minor ownership and rural ownership. Full ownership is where the owner has a property certificate issued by state authority, which is transferrable in the housing market. Partial ownership is where the owner does not have a state-issued property certificate. Partial property rights are usually acquired with housing purchased at prices subsidized by governments or enterprises. A partial property right gives a homeowner the right to use the property or to sell it after a certain period with restrictions on the treatment of capital gains. Minor homeownership refers to housing with limited property rights, built on collectively owned rural land and sold to buyers outside the collective ownership. Theoretically, this form of ownership is not officially recognized and thus illegal in China, although in practice it is a common phenomenon in urban villages and suburban areas. We construct 4 ownership binary dummies to represent the housing ownership. Third, in most cases, a household purchase the house with loan offered by bank or private. We denote the existence of a bank loan or private loan by a binary dummy. Moreover, we also use the proportion of housing loan to the housing assets to measure the degree of external financial reliance. Finally, with respects to the housing acquisition, generally, there are 4 kinds of acquisition methods including purchase with market price, purchase with low price, inherited or gifted, and self-built. Similarly, we generate 4 binary dummies to proxy the variable. Z represents the control variables consisting of age, gender, education, Hukou, risk attitude, the number of children. Table 1 defines all variables.

**Table 1:** Variable definitions.

Variable	Definition
Agricultural Entrepreneurship	1 for household engaging agricultural entrepreneurship; 0 otherwise.
Business Entrepreneurship	1 for household engaging business entrepreneurship; 0 otherwise.
House	1if a household own a house or more, 0 otherwise.
House number	The number of houses owned by a household.
House share	The ratio of housing assets to total assets
Partial ownership	1 for partial ownership, 0 otherwise.
Full ownership	1 for full ownership, 0 otherwise.
Minor ownership	1 for minor ownership, 0 otherwise.
Rural ownership	1 for rural ownership, 0 otherwise.
Bank loan	1 for a family with a home bank loan,0 otherwise.
Private loan	1 for a family with a home folk loan,0 otherwise.
Loan share	The proportion of housing loan to the housing assets
Low price house	1 if households purchase house at a price lower than the market price. 0 otherwise.
Market price house	1 if households acquire house at market price.0 otherwise.
Self-built house	1 for the self-built/expanded house, 0 otherwise.
Inherited or gifted house	1 if the house is inherited or gifted, 0 otherwise.
Age	The age of householder
Age^2	The square of the householder age
Gender	Male = 1; female = 0.
Education	Never attended school = 1; primary school education = 2; junior high school education = 3; high school education = 4; technical secondary school / vocational education = 5; junior college / vocational education = 6; bachelor’s degree = 7; master’s degree = 8; doctoral degree = 9.
Hukou	Urban registered residence = 1; Rural registered residence = 0.
Marital status	Married = 1, others = 0
Risk attitude	Unwilling to take any risk = 1; slightly lower risk, slightly lower return projects = 2; average risk, average return = 3; slightly higher risk and slightly higher return projects = 4; High risk and high return projects = 5.
Children number	The number of children a household has

### 4.3 Description

Table 2 statistically describes all variables in the specification by entrepreneurship type. As can be seen from this table, only 2.46% of households are engaged in agricultural entrepreneurship. 16.69% of households are engaged in business entrepreneurship. The average probability of owning houses was 97.58% for agricultural entrepreneurs and 91.55% for business entrepreneurs. Both agricultural and business entrepreneurs own more than one house. 3.68% of agricultural entrepreneurs and 9.46% of business entrepreneurs obtain housing loan from bank, and this number for the private loan is 12.23% and 29.38% respectively. The average proportion of housing liabilities to total assets is 7.73% for agricultural entrepreneurs and 8.62% for business entrepreneurs. These results indicate that business entrepreneurs are more dependent on the external borrowing than agricultural entrepreneurs when buying a house. In addition, both entrepreneurs are more likely to receive private finance than formal finance.

**Table 2:** Summary statistics.

Variables	Agricultural entrepreneurship (2.46%)		Business entrepreneurship (16.69%)	
	Mean	Standard error	Mean	Standard error
House	0.9758	0.1535	0.9155	0.2781
House number	1.1531	0.4607	1.4123	14.0887
House share	0.5544	0.285	0.6315	0.2785
Partial ownership	0.02799	0.1649	0.03525	0.1844
Full ownership	0.1762	0.4471	0.7346	0.4718
Minor ownership	0.00968	0.9791	0.01238	0.1106
Rural ownership	0.7056	0.3074	0.05881	0.2353
Bank loan	0.0368	0.18827	0.09462	0.2927
Private loan	0.1223	0.3277	0.2938	0.2853
Loan share	0.07726	0.6871	0.0862	0.5076
Low price house	0.1199	0.3249	0.2935	1.3374
Market price house	0.3395	0.4735	0.453	0.356
Self-built house	0.4313	0.4952	0.1942	0.3956
Inherited or gifted house	0.1067	0.3088	0.0481	0.2139
Age	56	12.4953	55	14.7491
Age^2	3292.172	1380.716	3249.751	1649.646
Gender	0.6269	0.4837	0.5285	0.4992
Education	2.6203	1.1013	3.5255	1.7186
Hukou	0.0985	0.0837	0.8955	0.4563
Marital status	0.9113	0.2842	0.8541	0.353
Risk attitude	3.2274	1.1413	4.0735	1.1742
Children number	1.0776	1.4692	0.8601	1.3363

## 5 Empirical results

### 5.1 The impact of housing wealth on entrepreneurship

Table 3 reports the estimation results of the impact of housing wealth on agricultural entrepreneurship. As shown in Column (1) of this table, the estimated coefficient of housing dummy is found to be negative and significance at 5% level, which suggests that housing owning lowers the possibility of participating agricultural entrepreneurial activities. In other words, households having own houses are less likely to engage in agricultural entrepreneurship. The result of column (2) shows that the coefficient of house number is positive and significant at 1% level, which means the more houses a household owns, the higher likelihood to start agricultural entrepreneurship. This is consistent with previous studies [16, 43]. Similarly, the result in column (3) shows that the estimated coefficient of house share is negative and significant at 1% level, indicating that the proportion of house asset to total assets has a negative impact on agricultural entrepreneurship. To summarize, higher housing wealth discourage householders to be entrepreneurs in agriculture. The main reasons to this fact may be that (1) urban householders almost have no chance to engage in agricultural entrepreneurship in China because they aren’t familiar with agricultural production and they aren’t allowed to have agricultural resources, such as arable land and forest. Thus, most agricultural entrepreneurs are rural households. Moreover, rural houses have low market value because they aren’t allowed to be traded, which gives birth to a low wealth effect of rural houses on entrepreneurship. Finally, richer rural householders are more likely to migrate to urban for the sake of better education for their children, more diverse lifestyle and better public services, and thus less likely to stay at rural areas, let alone starting an agricultural business.

**Table 3:** Results of housing wealth on agricultural entrepreneurship.

VARIABLES	(1)	(2)	(3)
	Agricultural entrepreneurship	Agricultural entrepreneurship	Agricultural entrepreneurship
House	-0.355**		
	(-0.148)		
House number		-0.135***	
		(-0.0473)	
House share			-0.520***
			(-0.133)
Age	0.00996	0.016	0.017
	(-0.0164)	(-0.017)	(-0.0227)
Age_2	-0.000106	-0.000161	-0.000159
	(-0.000155)	(-0.00016)	(-0.000213)
Gender	0.0541	0.0467	0.031
	(-0.0611)	(-0.0624)	(-0.0814)
Education	0.169***	0.160***	0.161***
	(-0.0242)	(-0.025)	(-0.0332)
Hukou	-0.0531	-0.0592	-0.0968
	(-0.06)	(-0.0611)	(-0.0843)
Marital status	0.210*	0.152	0.246
	(-0.115)	(-0.117)	(-0.169)
Risk attitude	-0.110***	-0.104***	-0.117***
	(-0.0226)	(-0.0231)	(-0.0292)
Children number	-0.0484*	-0.0451*	-0.0401
	(-0.0265)	(-0.0269)	(-0.0343)
Constant	-2.042***	-2.654***	-2.278***
	(-0.44)	(-0.447)	(-0.607)
Observations	10,229	9,975	5,861
R^2^	0.1141	0.1083	0.0987

Note:

*** p <0.01,

** p <0.05,

* p <0.1.

Robust standard deviations in parentheses.

As for the control variables, it can be seen from Table 2 that age and gender have no significant influence on agricultural entrepreneurship. Education, marital status and risk attitude have a significant positive influence on agricultural entrepreneurship. However, the number of children has a significant negative impact on agricultural entrepreneurship.

Table 4 reports the estimated results of the impact of homeownership on business entrepreneurship. From this table, we observe that the coefficient of house dummy is positive and significant at 1% level, which implies that owning house has a significant positive effect on business entrepreneurship. In short, houseowners are more likely to run their own business. The coefficient of house number is found to be positive and significant at 10% level. This positive effect suggests that housing investments can have wealth and collateral effects, and that the more houses a household owns, the higher the likelihood of engaging in business entrepreneurial activity [26, 28]. At last, we find that the coefficient of house share is also positive and significant at 1% level. This result can be interpreted as households with higher share of housing assets are more inclined to engage in business entrepreneurial activities. Taking all these results into consideration, we can point out that homeownership plays a positive role on the willingness of starting a new business. The wealthy and collateral effects of housing are speculated to be major contribution to this positive effect.

**Table 4:** Results of housing wealth on business entrepreneurship.

VARIABLES	(1)	(2)	(3)
	Business entrepreneurship	Business entrepreneurship	Business entrepreneurship
House	0.163***		
	(-0.0321)		
House number		0.0566*	
		(-0.0313)	
House share			0.388***
			(-0.0473)
Age	-0.00324	-0.00529	-0.00898
	(-0.00447)	(-0.0047)	(-0.00685)
Age_2	-0.000174***	-0.000149***	-0.000105
	(-4.23E-05)	(-4.42E-05)	(-6.39E-05)
Gender	0.143***	0.143***	0.110***
	(-0.0175)	(-0.0183)	(-0.0263)
Education	-0.0697***	-0.0655***	-0.112***
	(-0.00596)	(-0.00623)	(-0.00927)
Hukou	0.359***	0.352***	0.591***
	(-0.0216)	(-0.0221)	(-0.0308)
Marital status	0.200***	0.181***	0.108**
	(-0.0281)	(-0.0303)	(-0.0454)
Risk attitude	0.0726***	0.0717***	0.0320***
	(-0.00761)	(-0.00794)	(-0.0114)
Children number	-0.0480***	-0.0523***	-0.0560***
	(-0.00935)	(-0.00964)	(-0.0133)
Constant	-0.00986	0.186	1.237***
	(-0.119)	(-0.124)	(-0.188)
Observations	32,836	29,991	16,077
R^2^	0.2297	0.1701	0.2075

Note:

*** p <0.01,

** p <0.05,

* p <0.1.

Robust standard deviations in parentheses.

Regarding with the control variables, the results show no significant effect of age on business entrepreneurship. Male, urban, married, and risk-preferred householders have stronger business entrepreneurship, which is consistent with the results in Hurst and Lusardi (2004) [29]. better-educated households are less likely to involve in business entrepreneurship. Households with more children are associated with a lower likelihood of doing business entrepreneurial activities.

### 5.2 The impact of housing ownership on entrepreneurship

We go to investigate the effect of housing ownership on the family entrepreneurship. The results of the impact of the housing ownership on agricultural entrepreneurship are presented in Table 5 and the results for business entrepreneurship are reported in Table 6.

**Table 5:** Results of housing ownership on agricultural entrepreneurship.

VARIABLES	(1)	(2)	(3)	(4)
	Agricultural entrepreneurship	Agricultural entrepreneurship	Agricultural entrepreneurship	Agricultural entrepreneurship
Partial ownership	-0.209			
	(-0.192)			
Full ownership		0.0295		
		(-0.061)		
			0.0487	
			(-0.267)	
Rural ownership				-0.0852
				(-0.0939)
Age	0.00792	0.00791	0.00765	0.00798
	(-0.0163)	(-0.0163)	(-0.0163)	(-0.0163)
Age_2	-8.89E-05	-8.98E-05	-8.73E-05	-9.12E-05
	(-0.000154)	(-0.000154)	(-0.000154)	(-0.000154)
Gender	0.0567	0.0549	0.0558	0.0586
	(-0.0611)	(-0.0611)	(-0.0611)	(-0.0611)
Education	0.169***	0.167***	0.168***	0.167***
	(-0.0242)	(-0.0241)	(-0.0241)	(-0.0241)
Hukou	-0.0632	-0.0585	-0.0602	-0.0651
	(-0.0598)	(-0.06)	(-0.0598)	(-0.06)
Marital status	0.207*	0.205*	0.204*	0.202*
	(-0.115)	(-0.115)	(-0.115)	(-0.115)
Risk attitude	-0.109***	-0.109***	-0.108***	-0.108***
	(-0.0225)	(-0.0225)	(-0.0225)	(-0.0225)
Children number	-0.0479*	-0.0468*	-0.0469*	-0.0467*
	(-0.0265)	(-0.0265)	(-0.0265)	(-0.0265)
Constant	-2.318***	-2.325***	-2.313***	-2.304***
	(-0.425)	(-0.426)	(-0.425)	(-0.425)
Observations	10,229	10,229	10,229	10,229
R^2^	0.1358	0.1363	0.1351	0.1344

Note:

*** p <0.01,

** p <0.05,

* p <0.1.

Robust standard deviations in parentheses.

**Table 6:** Results of housing ownership on business entrepreneurship.

VARIABLES	(1)	(2)	(3)	(4)
	Business entrepreneurship	Business entrepreneurship	Business entrepreneurship	Business entrepreneurship
Partial ownership	-0.0625			
	(-0.0466)			
Full ownership		0.0627***		
		(-0.0181)		
Minor ownership			-0.0236	
			(-0.0738)	
Rural ownership				0.000805
				(-0.0359)
Age	-0.00214	-0.00183	-0.00222	-0.00223
	(-0.00447)	(-0.00447)	(-0.00447)	(-0.00447)
Age_2	-0.000181***	-0.000185***	-0.000181***	-0.000180***
	(-4.23E-05)	(-4.24E-05)	(-4.23E-05)	(-4.23E-05)
Gender	0.145***	0.144***	0.145***	0.145***
	(-0.0175)	(-0.0175)	(-0.0175)	(-0.0176)
Education	-0.0683***	-0.0702***	-0.0683***	-0.0683***
	(-0.00595)	(-0.00598)	(-0.00595)	(-0.00598)
Hukou	0.347***	0.342***	0.347***	0.346***
	(-0.0215)	(-0.0216)	(-0.0215)	(-0.0215)
Marital status	0.214***	0.212***	0.214***	0.214***
	(-0.0279)	(-0.0279)	(-0.0279)	(-0.0279)
Risk attitude	0.0733***	0.0728***	0.0733***	0.0733***
	(-0.00761)	(-0.00761)	(-0.00761)	(-0.00761)
Children number	-0.0485***	-0.0482***	-0.0485***	-0.0485***
	(-0.00935)	(-0.00935)	(-0.00935)	(-0.00935)
Constant	0.0887	0.0637	0.0888	0.0886
	(-0.117)	(-0.118)	(-0.117)	(-0.118)
Observations	32,843	32,843	32,843	32,843
R^2^	0.1872	0.1881	0.1870	0.1864

Note:

*** p <0.01,

** p <0.05,

* p <0.1.

Robust standard deviations in parentheses.

Looking at Table 5, all coefficients concerning housing ownerships are found to be insignificant, indicating that housing ownerships have no significant effect on the intention of engaging in the agricultural entrepreneurship. Furthermore, as shown in Table 6, We find that only full ownership carries a positive and significant coefficient, implying that households owning houses with full ownership are more likely to start a new business entrepreneurship. the possible reason to this result lie in that houses with full ownership have much higher market value that any other forms of property rights, leading to a higher positive wealthy and collateral effect of housing on business entrepreneurial likelihood. Except for full ownership, all other estimated coefficients of housing ownerships are still found to be insignificant, which suggest that these housing ownerships have insignificant impact on business entrepreneurial probability. In terms of control variables, we find consistent results with the previous analysis in both Tables 5 and 6.

### 5.3 The impact of housing loan on entrepreneurship

We now exam the effects of housing loan on entrepreneurship. Table 7 reports the estimated results of the impact of housing loan on agricultural entrepreneurship. As displayed in this table, the bank loan is associated with a positive coefficient which is significant at 10% level, implying that bank loan has a positive impact on agricultural entrepreneurship. householders with housing loan from bank are more likely to be agricultural entrepreneurs. We try to explain this finding as follows. First, farmers are most likely to start businesses in agriculture. However, housing loans from banks only apply for urban houses in China. Farmers couldn’t mortgage rural houses from bank, because they are unable to trade in the real estate market. As a result, farmers are sure to have purchased urban house if they have housing bank loan. Second, the pressure caused by the repayment of housing bank loan will stimulate the intention of engaging in agricultural entrepreneurship for rural householders, because the lower return of regular agricultural production brings about higher risk of debt default.

**Table 7:** Results of housing loan on agricultural entrepreneurship.

VARIABLES	(1)	(2)	(3)
	Agricultural entrepreneurship	Agricultural entrepreneurship	Agricultural entrepreneurship
Bank loan	0.198*		
	(-0.119)		
Private loan		-0.291***	
		(-0.105)	
Loan share			-0.162*
			(-0.172)
Age	0.0204	0.0195	0.0194
	(-0.0173)	(-0.0174)	(-0.0225)
age_2	-0.000201	-0.000199	-0.000189
	(-0.000164)	(-0.000165)	-0.000211
Gender	0.0505	0.0467	0.0672
	(-0.0627)	(-0.0629)	(-0.0804)
Education	0.164***	0.168***	0.170***
	(-0.0252)	(-0.025)	(-0.0329)
Hukou	-0.0468	-0.0419	-0.0783
	(-0.0617)	(-0.0619)	(-0.0836)
Marital status	0.14	0.15	0.243
	(-0.117)	(-0.117)	(-0.166)
Risk attitude	-0.107***	-0.110***	-0.123***
	(-0.0231)	(-0.0232)	(-0.0289)
Children number	-0.0498*	-0.0528*	-0.0386
	(-0.0273)	(-0.0276)	(-0.034)
Constant	-2.616***	-2.538***	-2.608***
	(-0.45)	(-0.452)	(-0.598)
Observations	9,940	9,926	5,852
R^2^	0.1063	0.1057	0.0991

Note:

*** p <0.01,

** p <0.05,

* p <0.1.

Robust standard deviations in parentheses.

Column (2) and column (3) shows opposite results to column (1). the estimated coefficient of both private loan and loan share are negative and significant, meaning that both private loan and loan share has significant negative effects on agricultural entrepreneurship. We may ascribe the negative effects of private loan and loan share on agricultural entrepreneurship to the fact that private loans have higher interest rates than bank loan and shorter repayment terms, resulting in a higher financial pressure to the borrowers. Meanwhile, higher loan share obviously leads to a stronger financial pressure. In order to make repayment on time, the debtors are less likely to engage in agricultural entrepreneurial activities which has high risks and long return period.

Table 8 reports the estimation results of the impact of housing loan on business entrepreneurship. We can know from the table that the coefficients of bank loan, private loan and loan share are found to be positive and significant, indicating that housing loan has a significant negative effect on the desire of involving in business entrepreneurship. That is to say householders having housing loans and higher loan share are less likely to start a new business. The reason of this finding is intuitive. The entrepreneurial enthusiasm suffers from the pressure caused by housing debt. This may be because the more housing debt a household owes, the greater the pressure to repay, the stronger the liquidity constraint faced by the household, and the deeper the household’s risk aversion, thus further reducing the probability that the household will engage in entrepreneurial activity [44].

**Table 8:** Results of housing loan on business entrepreneurship.

VARIABLES	(1)	(2)	(3)
	Business entrepreneurship	Business entrepreneurship	Business entrepreneurship
Bank loan	-0.0685**		
	(-0.0292)		
Private loan		-0.279***	
		(-0.0335)	
Loan share			-0.206***
			(-0.0609)
Age	-0.00492	-0.0051	-0.00452
	(-0.00472)	(-0.00473)	(-0.0066)
Age_2	-0.000152***	-0.000157***	-0.000167***
	(-4.45E-05)	(-4.46E-05)	(-6.14E-05)
Gender	0.148***	0.148***	0.179***
	(-0.0184)	(-0.0184)	(-0.0253)
Education	-0.0680***	-0.0692***	-0.0885***
	(-0.00636)	(-0.00628)	(-0.00893)
Hukou	0.354***	0.348***	0.410***
	(-0.0222)	(-0.0222)	(-0.029)
Marital status	0.177***	0.185***	0.174***
	(-0.0304)	(-0.0304)	(-0.0437)
Risk attitude	0.0700***	0.0713***	0.0575***
	(-0.00798)	(-0.00799)	(-0.011)
Children number	-0.0518***	-0.0529***	-0.0454***
	(-0.00969)	(-0.00972)	(-0.0129)
Constant	0.174	0.232*	0.252
	(-0.125)	(-0.125)	(-0.178)
Observations	29,799	29,753	16,046
R^2^	0.1659	0.1645	0.2009

Note:

*** p <0.01,

** p <0.05,

* p <0.1.

Robust standard deviations in parentheses.

### 5.4 The impact of housing acquisition on entrepreneurship

Table 9 reports the results of the influence of different house acquisition methods on agricultural entrepreneurship using the Probit model. As indicated in this table, the coefficient of self-built house is positive and significant at 5% level, suggesting that households who build their own house enjoy a higher chance to become agricultural entrepreneurs. As stated earlier, only rural households are permitted build their own houses on homestead. Thus, households who have self-built houses can be identified as rural households. Rural households are certain to have a higher possibility to be agricultural entrepreneurs, not to speak of rural households who have spent money on the construction of their own houses.

**Table 9:** Results of housing acquisition on agricultural entrepreneurship.

VARIABLES	(1)	(2)	(3)	(4)
	Agricultural entrepreneurship	Agricultural entrepreneurship	Agricultural entrepreneurship	Agricultural entrepreneurship
Low price house	-0.156			
	(-0.16)			
Market price house		-0.109		
		(-0.0935)		
Self-built house			0.197**	
			(-0.0902)	
Inherited or gifted house				-0.071
				(-0.133)
Age	0.0265	0.0254	0.0269	0.026
	(-0.0205)	(-0.0204)	(-0.0206)	(-0.0204)
Age_2	-0.000319	-0.000315	-0.000321	-0.000319
	(-0.000199)	(-0.000198)	(-0.0002)	(-0.000198)
Gender	0.129*	0.127*	0.114	0.132*
	(-0.077)	(-0.077)	(-0.0775)	(-0.0768)
Education	-0.000115	0.00485	0.0147	-0.00375
	(-0.0257)	(-0.0264)	(-0.0269)	(-0.0255)
Hukou	0.469***	0.457***	0.410***	0.478***
	(-0.0824)	(-0.0838)	(-0.0868)	(-0.0819)
Marital status	-0.00431	0.00734	0.0027	-0.000486
	(-0.12)	(-0.12)	(-0.121)	(-0.12)
Risk attitude	-0.0761**	-0.0764**	-0.0770***	-0.0750**
	(-0.0298)	(-0.0298)	(-0.0299)	(-0.0298)
Children number	-0.0259	-0.0246	-0.0313	-0.0241
	(-0.039)	(-0.0389)	(-0.0391)	(-0.0389)
Constant	-2.817***	-2.774***	-2.972***	-2.796***
	(-0.519)	(-0.517)	(-0.526)	(-0.518)
Observations	14,861	14,905	14,905	14,905
R^2^	0.1921	0.1977	0.1979	0.1986

Note:

*** p <0.01,

** p <0.05,

* p <0.1.

Robust standard deviations in parentheses.

The rest relevant variables including low price house, market price house and inherited or gifted house are associated with negative but insignificant coefficients, suggesting an insignificant effect of them on agricultural entrepreneurship. This result could be explained by the reasons elaborated in section 5.1.

Table 10 lists the estimation results of the impact of housing acquisition methods on business entrepreneurship. We can observe from column (1) and column (4), the coefficients of low-price house and inherited or gifted house are found to be negative and significant at 1% level, indicating that both buying houses at below-market price and acquiring houses through inheritance or gift suppress the intention of engaging in business entrepreneurship. The negative effect of low-price houses may relate to the low market value of low-price houses, and consequently the low wealth effect of this kind of houses. What’s more, the low-price houses have no collateral effect because they couldn’t be used as the collateral. The inhibitory effect of inherited or gifted houses could be traced to the lower pressure caused by the housing. Households who acquire houses without paying any prices normally have a lower motivation to run a new business.

**Table 10:** Results of housing acquisition on business entrepreneurship.

VARIABLES	(1)	(2)	(3)	(4)
	Business entrepreneurship	Business entrepreneurship	Business entrepreneurship	Business entrepreneurship
Low price house	-0.339***			
	(-0.0458)			
Market price house		0.170***		
		(-0.0283)		
Self-built house			0.0569	
			(-0.0299)	
Inherited or gifted house				-0.180***
				(-0.043)
Age	-0.00104	-0.002	-0.00156	-0.00158
	(-0.00657)	(-0.00653)	(-0.00653)	(-0.00653)
Age_2	-0.000166***	-0.000173***	-0.000175***	-0.000176***
	(-6.28E-05)	(-6.24E-05)	(-6.24E-05)	(-6.24E-05)
Gender	0.104***	0.126***	0.109***	0.112***
	(-0.0256)	(-0.0256)	(-0.0257)	(-0.0255)
Education	-0.0388***	-0.0630***	-0.0433***	-0.0509***
	(-0.00844)	(-0.00868)	(-0.00884)	(-0.00835)
Hukou	0.315***	0.255***	0.315***	0.305***
	(-0.0332)	(-0.0338)	(-0.0348)	(-0.0332)
Marital status	0.168***	0.164***	0.174***	0.168***
	(-0.041)	(-0.0408)	(-0.0408)	(-0.0408)
Risk attitude	0.0818***	0.0807***	0.0823***	0.0813***
	(-0.011)	(-0.011)	(-0.011)	(-0.011)
Children number	-0.0610***	-0.0518***	-0.0564***	-0.0562***
	(-0.0141)	(-0.0141)	(-0.0141)	(-0.0141)
Constant	0.0284	0.0606	0.0332	0.105
	(-0.17)	(-0.1690	(-0.171)	(-0.169)
Observations	14,861	14,905	14,905	14,905
R^2^	0.2133	0.2161	0.2175	0.2166

Note:

*** p <0.01,

** p <0.05,

* p <0.1.

Robust standard deviations in parentheses.

Column (2) show that the coefficients of market price house is positive and significant, implying that market price house imposes a promoting influence on the likelihood of being business entrepreneurs. This result can be illustrated by the wealth and collateral effects born with normal housing. At last, the insignificant effect of self-built house on business entrepreneurship is discovered in column (3), which can be explained by the mismatch between rural households and business operations.

### 5.5 Endogeneity

The reliability of the benchmark results may suffer from the endogenous problem caused by housing. The endogeneity mainly comes from the reverse causality between housing and entrepreneurship. The housing condition of a household isn’t only a factor impacting entrepreneurship, but may also be affected by their entrepreneurship [16]. In order to address the endogeneity, we employ the lag of the core independent variables, which is the observations of housing condition in 2013, as the instrumental variable (IV). This instrument variable was chosen because it meets two conditions as a good instrument variable including the exogeneity of instrumental variable and the strong correlation between instrumental and endogenous variable. At first, the housing condition in 2013 has nothing to do with the current disturbances, so that it meets the requirement of exogeneity. Second, there is a time series relationship between the housing in 2013 and that in 2015, so that they are necessarily related. At beginning, we execute Hausman tests in order to identify the endogeneity of four proxies for housing. The rejection of the null hypothesis indicates the existence of endogenous problem. It is found through tests that endogeneity of housing exists in four regressions as shown in Table 11.

**Table 11:** Results of IV regressions.

Variables	(1)	(2)	(3)	(4)
	Business entrepreneurship	Business entrepreneurship	Business entrepreneurship	Business entrepreneurship
House number	0.154***			
	(0.0266)			
Private loan		-0.0051***		
		(0.00124)		
Self-built house			1.693***	
			(0.341)	
Inherited or gifted house				-0.819***
				(1.545)
Controls	Controlled	Controlled	Controlled	Controlled
N	12259	13128	19093	19093
First-stage F value	70.18	13.86	55.15	13.61
DWH	49.5418	20.0127	28.0102	53.0082

Note:

*** p <0.01,

** p <0.05,

* p <0.1.

Standard deviations in parentheses.

Table 11 presents the results of the two-stage regression using instrumental variables using with ivprobit command. Since the exogeneity is naturally satisfied when using the lagged variables as IVs, what’s left to do is the identification of weak instruments. A general rule of thumb requires an F value in the first stage at least 10 to expel the concern of weak instruments [45]. As exhibited in Table 11, F-values of the first stage are in all columns are larger than the critical value, suggesting there is no concern of weak instruments in the regressions.

We can observe from the table that the coefficients of housing-related variables remain unchanged, which means that after addressing the concern of endogeneity, the results reached are still in line with the benchmark analysis, which verifies the results of our research.

### 5.6 Robustness checks

From the results obtained from benchmark and endogeneity study, we have reached some findings between housing and family entrepreneurship. However, we should be cautious about the safety of this findings, which needs to more evidences. Therefore, in this section, we offer some robustness checks to the benchmark results using an alternative regression method-Logit model. From Tables 12 to 15, we find the consistent results with the benchmark results after using a different estimation method, which illustrates the results of our study are robust.

**Table 12:** Robust results of housing wealth on household entrepreneurship(logit).

VARIABLES	(1)	(2)	(3)	(4)	(5)	(6)
	Agricultural entrepreneurship	Agricultural entrepreneurship	Agricultural entrepreneurship	Business entrepreneurship	Business entrepreneurship	Business entrepreneurship
House	-0.340**			0.166***		
	(-0.147)			(-0.0321)		
House number		0.146***			0.000515	
		(-0.047)			(-0.000556)	
House share			-0.542***			-1.395***
			(-0.132)			(-0.0473)
Controls	Controlled	Controlled	Controlled	Controlled	Controlled	Controlled
Constant	-2.323***	-2.875***	-2.537***	-0.333***	-0.138	1.106***
	(-0.439)	(-0.445)	(-0.605)	(-0.118)	(-0.124)	(-0.187)
Observations	10,211	9,958	5,848	32,802	29,960	16,060

Note:

*** p <0.01,

** p <0.05,

* p <0.1.

Robust standard deviations in parentheses.

**Table 13:** Robust results of housing ownership on entrepreneurship(logit).

VARIABLES	(1)	(2)	(3)	(4)	(5)	(6)	(7)	(8)
	Agricultural entrepreneurship	Agricultural entrepreneurship	Agricultural entrepreneurship	Agricultural entrepreneurship	Business entrepreneurship	Business entrepreneurship	Business entrepreneurship	Business entrepreneurship
Partial ownership	-0.203				-0.0574			
	(-0.191)				(-0.0466)			
Full ownership		0.0244				0.0652***		
		(-0.0606)				(-0.018)		
Minor ownership			0.0906				-0.0186	
			(-0.26)				(-0.0737)	
Rural Ownership				-0.0865				0.0048
				-0.0935				(-0.0359)
Controls	Controlled	Controlled	Controlled	Controlled	Controlled	Controlled	Controlled	Controlled
Constant	-2.587***	-2.593***	-2.582***	-2.573***	-0.236**	-0.261**	-0.236**	-0.237**
	-0.425	-0.425	-0.424	-0.425	-0.117	-0.117	-0.117	-0.117
Observations	10,211	10,211	10,211	10,211	32,809	32,809	32,809	32,809

Note:

*** p <0.01,

** p <0.05,

* p <0.1.

Robust standard deviations in parentheses.

**Table 14:** Robust results of housing loan on entrepreneurship(logit).

VARIABLES	(1)	(2)	(3)	(4)	(5)	(6)
	Agricultural entrepreneurship	Agricultural entrepreneurship	Agricultural entrepreneurship	Business entrepreneurship	Business entrepreneurship	Business entrepreneurship
Bank loan	0.212*			0.0743**		
	(-0.118)			(-0.0292)		
Private loan		-0.268***			-0.276***	
		(-0.104)			(-0.0335)	
Loan share			-0.149			-0.205***
			(-0.165)			(-0.06090
Controls	Controlled	Controlled	Controlled	Controlled	Controlled	Controlled
Constant	-2.836***	-2.767***	-2.936***	-0.139	-0.0825	-0.00744
	(-0.4490	(-0.451)	(-0.595)	(-0.124)	(-0.124)	(-0.177)
Observations	9,923	9,910	5,839	29,768	29,723	16,029

Note:

*** p <0.01,

** p <0.05,

* p <0.1.

Robust standard deviations in parentheses.

**Table 15:** Robust results of housing acquisition on entrepreneurship(logit).

VARIABLES	(1)	(2)	(3)	(4)	(5)	(6)	(7)	(8)
	Agricultural entrepreneurship	Agricultural entrepreneurship	Agricultural entrepreneurship	Agricultural entrepreneurship	Business entrepreneurship	Business entrepreneurship	Business entrepreneurship	Business entrepreneurship
Low price house	-0.148				-0.347***			
	(-0.16)				(-0.0458)			
Market price house		-0.0996				0.173***		
		(-0.0933)				(-0.0283)		
Self-built house			0.187**				0.0594**	
			(-0.0898)				-0.03	
Inherited house				-0.0783				-0.184***
				(-0.133)				(-0.0429)
Controls	Controlled	Controlled	Controlled	Controlled	Controlled	Controlled	Controlled	Controlled
Constant	-2.966***	-2.926***	-3.118***	-2.940***	-0.373**	-0.324*	-0.366**	-0.285*
	(-0.5170	(-0.515)	(-0.525)	(-0.516)	(-0.169)	(-0.168)	(-0.17)	(-0.168)
Observations	14,846	14,890	14,890	14,890	14,846	14,890	14,890	14,890

Note:

*** p <0.01,

** p <0.05,

* p <0.1.

Robust standard deviations in parentheses.

## 6 Conclusions

The purpose of this article is to investigate the impact of housing, which is measured with housing wealth, housing ownership, housing loan and housing acquisition on the agricultural and business entrepreneurship by using the data from China Household Finance Survey (CHFS) in 2015. The results shows that (1) housing wealth has a negative effect on agricultural entrepreneurship but a positive effect on business entrepreneurship. Households owning higher housing wealth are less likely to become agricultural entrepreneurs, but more likely to start a new business. (2) Households with full-owned housing enjoy a higher likelihood to become business entrepreneurs. Other ownerships have no relation with entrepreneurship. (3) Housing loans wherever obtained have a significant negative impact on family entrepreneurship. households with more housing loans are less likely to engage in entrepreneurial activities. One exception to this result is that bank loan increases the probability of being agricultural entrepreneurs. (4) The effects of housing acquisition are more complex. The positive effect of self-built houses on agricultural entrepreneurship indicates that households who build their own houses are associated with a higher agricultural entrepreneurship. However, low-price house and inherited or gifted house play a negative role on the business entrepreneurship. While households who buy houses at market price are more inclined to be business entrepreneurs. All results are testified to be reliable after addressing potential endogenous problem and executing some robustness checks.

Our study may carry some policy implications to promote the entrepreneurship in China. First, the negative effect of housing wealth on agricultural entrepreneurship suggests that more efforts should be put on encouraging agricultural entrepreneurship by government. Next, given the negative effects of housing loan on entrepreneurship, the housing price should be controlled to ameliorate the financial pressure caused by buying a house. Meanwhile, the more formal financial resources should be allocated to housing market to avoid the higher cost of private loan. Finally, as suggested by the negative effect of low-price housing and positive impact of full ownership on business entrepreneurship, the housing reform should be carried out to a deeper degree in order to eliminate houses without full ownership, and thus form a unified real estate market.
