# Expression of Concern: Housing investment and family entrepreneurship: Evidence from China

**DOI:** 10.1371/journal.pone.0343665

**Published:** 2026-02-25

**Authors:** The PLOS One Editors

After this article [1] was published, the following issues were identified:

- Potential non-compliance with the PLOS Authorship policy.
- The specific datasets and sources of data used in this study are not provided with the article or described in sufficient detail to be reproducible.
- A lack of supporting citations for statements in the Introduction section.
- The inclusion of causal statements in the Abstract that are not supported by the article’s data and analyses.
- A lack of discussion of the study’s limitations, including the absence of key influencing factors.

The first author declared that they are unable to share the data due to data sharing restrictions imposed by the data owner, the China Household Finance Survey and Research Center of Southwestern University of Finance and Economics (http://chfs.swufe.edu.cn). The authors provided documentation to the Editors to confirm that they had permission to use the data set.

Regarding data extraction and preprocessing, the first author stated that they extracted household samples from the CHFS 2015 database from 29 provinces across the country, focusing on screening variables related to housing investment (e.g., housing holdings, type of ownership, loan status, etc.) and entrepreneurial behaviors (agricultural entrepreneurship, commercial entrepreneurship), and processed them through the Stata software; they excluded samples with missing and extreme values of key variables, resulting in a sample of 32,986 valid households.

The first author acknowledged the lack of citations in the Introduction section, and provided supporting references. The Editors were unable to confirm that all provided references support the corresponding statements, and they consider this issue unresolved.

The first author provided a rationale for claims regarding causation; however, the editors remain concerned that the study design was not sufficient to confirm causation rather than correlation.

The first author acknowledged the absence of discussion of the study’s limitations, and provided the following text to address this issue, which the Editors consider resolved: “First, in terms of the division of entrepreneurship types, entrepreneurship is simply categorized into agricultural entrepreneurship and commercial entrepreneurship, and this dichotomy fails to comprehensively cover the rich and diverse forms of entrepreneurship, and emerging forms of entrepreneurship such as high-tech entrepreneurship, social entrepreneurship, and digital entrepreneurship are not included in the scope of the study. Second, there are obvious limitations in data timeliness, as the study mainly uses data from the 2015 China Household Finance Survey (CHFS), which is a long time ago, during which China’s housing market and entrepreneurial environment have changed to some extent. Finally, this study did not directly incorporate the household income variable, which may underestimate or overestimate the independent impact of housing due to the correlation between income and housing assets.”

In light of the cumulative issues and concerns which were not resolved in our discussions with the authors, the *PLOS One* Editors issue this Expression of Concern. Readers are advised to interpret the article [1] with caution.
